# Antitrans Policy Environment and Depression and Anxiety Symptoms in Transgender and Nonbinary Adults

**DOI:** 10.1001/jamanetworkopen.2024.31306

**Published:** 2024-08-22

**Authors:** Arjee Restar, Eric K. Layland, Landon Hughes, Emerson Dusic, Ruby Lucas, Audren J. K. Bambilla, Aleks Martin, Alic Shook, Baer Karrington, Deborah Schwarz, Genya Shimkin, Vanessa Grandberry, Xero Xanadu, Carl G. Streed, Don Operario, Kristi E. Gamarel, Trace Kershaw

**Affiliations:** 1 Department of Epidemiology, School of Public Health, University of Washington, Seattle; 2 Department of Health Systems and Population Health, School of Public Health, University of Washington, Seattle; 3 Department of Social and Behavioral Sciences, Yale University School of Public Health, New Haven, Connecticut; 4 Weitzman Institute, Moses Weitzman Health System, Washington, DC; 5 Department of Human Development and Family Sciences, University of Delaware, Newark; 6 Department of Epidemiology, Harvard T. H. Chan School of Public Health, Boston, Massachusetts; 7 Public Health–Seattle and King County, Seattle, Washington; 8 College of Nursing, Seattle University, Seattle, Washington; 9 Department of Pediatrics, Seattle Children’s Hospital and University of Washington, Seattle; 10 Section of General Internal Medicine, Boston University Chobanian and Avedisian School of Medicine, Boston, Massachusetts; 11 Department of Behavioral, Social, and Health Education Sciences, Emory University Rollins School of Public Health, Atlanta, Georgia; 12 Department of Health Behavior and Health Education, University of Michigan School of Public Health, Ann Arbor

## Abstract

**Importance:**

With the increasing legislation restricting health care access for transgender and nonbinary (trans) populations in recent years, there has been limited research on how awareness of and concerns about legislative restrictions and protections influence mental health outcomes.

**Objective:**

To examine whether awareness of and concerns about the current policy environment regarding trans individuals are associated with depression and anxiety symptoms among trans adults.

**Design, Setting, and Participants:**

This study uses cross-sectional data collected between March and April 2023 from the Washington Priority Assessment in Trans Health (PATH) Project, an online study designed by, with, and for trans communities. All participants were trans adults, aged 18 years or older, living in Washington state.

**Exposure:**

Awareness and concerns about the antitrans policy environment.

**Main Outcomes and Measures:**

The primary outcomes were depression and anxiety symptoms, assessed via the Patient Health Questionnaire–4. A series of multivariable regression models was used to assess the association between awareness and concerns about the antitrans policy environment and depression and anxiety symptoms. Models were adjusted for covariates, including demographics, social marginalization, and health care experiences.

**Results:**

A total of 797 participants (653 women [81.93%]; 455 aged 18-29 years [57.09%]) were included. The majority screened positive for current depression (689 individuals [86.45%]) and anxiety (686 individuals [86.07%]) symptoms. Trans individuals who were concerned or worried about their rights being taken away (vs not) had significantly higher odds of current depression symptoms (adjusted odds ratio [aOR], 1.66; 95% CI, 1.08-2.54), as well as current anxiety symptoms (aOR, 2.67; 95% CI, 1.63-4.36). Those who knew (vs did not know) about state-level protective legislation had significantly lower odds of current depression symptoms (aOR, 0.44; 95% CI, 0.28-0.67), as well as current anxiety symptoms (aOR, 0.11; 95% CI, 0.04-0.25). When examining interaction effect estimates, trans individuals who correctly knew about the protective policies and were not worried about having their rights taken away reported the lowest odds of depression and anxiety.

**Conclusions and Relevance:**

The findings of this cross-sectional study are consistent with research elucidating the negative mental health consequences of policies limiting health care access and provide insights into informing policies and interventions that target trans populations’ worsened mental health outcomes as a result of antitrans legislation.

## Key Points

**Question:**

Is awareness of current policies addressing access to health care for transgender and nonbinary (trans) populations associated with depression and anxiety?

**Findings:**

In this cross-sectional study of 797 trans individuals living in Washington State, concerns about trans rights being taken away were associated with significantly higher odds of depression and anxiety symptoms. Individuals with accurate knowledge of state-level protective legislation had lower odds of depression and anxiety.

**Meaning:**

In a policy environment with increasing legislation aimed at eroding trans rights and access to care, interventions that increase awareness and accurate knowledge of policies that protect access to trans health care could help address distress around losing trans rights and may mitigate the negative impact of nationwide antitrans policies on mental health outcomes in trans adults.

## Teaser

This cross-sectional study examines whether awareness of and concerns about legislation restricting health care access for transgender and nonbinary people is associated with symptoms of depression and anxiety symptoms in transgender adults.

## Introduction

Transgender and nonbinary (hereafter, *trans*) populations experience high levels of adverse mental health outcomes, particularly depression and anxiety, compared with cisgender populations. In the US, prevalence estimates are as high as 84% for depression and 68% for anxiety among trans adults.^1,2^ Although studies^3,4,5,6,7^ have noted that access to medical gender-affirming services (eg, gender-affirming hormone therapy) can help alleviate these adverse outcomes, barriers to accessing both gender-affirming and mental health services continue to exist. Documented barriers to accessing health services include lack of, or inadequate, insurance coverage, inconsistent availability of trans-competent clinicians, health care mistreatment, and fear of stigma and discrimination.^8,9,10,11^

Trans individuals experience marginalization within the sociopolitical environment, with hundreds of pieces of legislation restricting their rights. In the last few years, states within the US have advanced a record number of bills targeting the restriction of lesbian, gay, bisexual, trans, and other queer protections and rights; as of June 2024, more than 598 bills across 43 states had been introduced, with 43 passed specific to targeting trans people’s rights.^12,13,14^ These bills include barring access to gender-affirming care for youth, updating name and gender marker in records, using correct name and pronouns, and curriculum around lesbian, gay, bisexual, trans, and other queer, Black, Asian American and Pacific Islander, and Indigenous histories in schools.^12,13,14^

Conversely, in Washington State, where this study is situated, several measures have been taken to protect trans rights. For example, the Washington Law Against Discrimination prohibits discrimination against anyone on the basis of their gender expression or identity in places of public accommodation, work, schools, housing, insurance transactions, and credit transactions.^15,16^ In 2021, the Gender Affirming Treatment Act was passed to ensure insurance companies could not deny coverage of gender-affirming treatments on the basis of considering them to be cosmetic.^17^ Furthermore, in response to the increasing antitrans legislation in 2023, Washington passed 5 new bills in the legislative session that protect trans youth in homeless shelters, individual privacy during name change procedures, health data accessibility, trans youth from discrimination or bullying in schools, and trans individuals’ rights to accessing gender-affirming care and reproductive health services.^16,17^ Despite protective legislative efforts, adverse mental health outcomes remain. According to the Trevor Project’s 2022 report,^18^ 63% of trans youth in Washington reported depression symptoms and 76% reported anxiety symptoms.

Although the existing sociopolitical environment around antitrans legislation has been shown to negatively impact mental health,^19,20^ there has been limited research elucidating the association between understanding of trans legislation and mental health outcomes. We hypothesize a direct association between awareness of negative trans health care policies and worse mental health of trans adults, while also considering how the extent to which trans people are aware of existing supportive legislative measures may be a protective factor in buffering the impact of an antitrans sociopolitical environment on mental health outcomes. The purpose of this study is to describe how policy awareness and concerns may be linked to current depression and anxiety symptoms in a sample of trans adults in Washington State.

## Methods

This cross-sectional study follows the Strengthening the Reporting of Observational Studies in Epidemiology (STROBE) reporting guidelines for cross-sectional analyses.^21^ The University of Washington Institutional Review Board Human Subject Division reviewed and approved all study procedures and materials. All enrolled participants provided electronic informed consent and consent for publication, after informing them about the study purpose and their rights to confidentiality, privacy, and withdrawal from the study.

### Participants and Setting

Data come from the Washington Priority Assessment in Trans Health (PATH) Project, a community-informed statewide health and wellness study with a cross-sectional survey developed for, by, and with trans Washington residents. Analyses focused on survey domains relevant to antitrans policy and mental health outcomes. Eligibility criteria were age 18 years or older, transgender or nonbinary identity, current residence in Washington, and willingness to provide electronic consent. Recruitment was via community-engaged approaches (eg, sampling via local organizations and community listservs, support groups, word-of-mouth, and social media platforms like Facebook and X [formerly Twitter]). For gender identity, we followed eligibility criteria based on a 2-step method^22^: first, we specifically asked individuals their gender (ie, “What is your gender identity?”) with the response options including (1) woman (including trans women), (2) man (including trans men), or (3) nonbinary (including gender nonconforming). The second question asked about sex assigned at birth, with response options of female, male, or intersex or a sex that is not exclusively male or female.

### Community Engagement

This project was implemented and launched with principles and practices of community engagement, discussed in detail elsewhere.^23^ In brief, the research investigative team assembled the Transgender Scientist and Stakeholder Advisory Board (TSSAB) before the launch of the study to ensure community input was incorporated from the beginning of the project. TSSAB members comprised trans individuals with diverse demographic characteristics, trans health expertise, and lived experiences as stakeholders, scientists, and community leaders and members in Washington. TSSAB members met monthly and were equitably part of the investigative team, such that project decisions across all parts of the survey design, including the selection of survey topics, framing of survey questions, as well as screening and recruitment approaches, were guided by TSSAB input.

### Researchers’ Positionality

In addition to an all-trans TSSAB, the investigative team is trans-led, and the coauthors of this manuscript are majority trans and queer researchers. This team structure allowed trans scholars to lead and guide the approach of researching salient topics within trans health and health policy while having cisgender investigators and scholars leverage resources, expertise, and labor in designing and executing studies while centering trans voices.^24^

### Measures

Survey topics and questionnaires were independently codeveloped by TSSAB members and trans-led community-based partners on the basis of priority health issues and needs of local trans communities. The survey was piloted to meet best-practice survey techniques, including minimizing survey fatigue (maximum duration, 25-30 minutes), enabling an automatic save setting to allow participants to return to the survey using the same device, and turning on a survey programming feature that prevents multiple responses from the same user.

### Exposure

To assess awareness of and concerns about the antitrans policy environment (as main exposure variables), we asked participants about their awareness of state-level hate crime policy protection, with the following knowledge-based item: “There is a transgender rights law in my state that protects people from hate crimes based on their gender identity/expression,” with response options as true, false, do not know, or not sure (the correct response is true).^15^ Participants’ responses were categorized as knew incorrectly, knew correctly, or did not know or unsure if they indicated that they were not aware or unsure about the policy. To assess concerns about antitrans policies, participants reported whether “[they were] worried that politicians where [they] live will pass laws that take away transgender people’s rights,” with responses coded as true (worried) or false (not worried).

### Outcomes

We used the 4-item Patient Health Questionnaire to measure depression and anxiety symptoms.^25^ Responses had acceptable Cronbach α reliability (α = 0.60). Summed scores of 3 or more indicated positive screenings for depression symptoms (items 1 and 2) and for anxiety (items 3 and 4) symptoms.

### Covariates

Sociodemographic characteristics collected included age, gender identity, race and ethnicity, sexual orientation, education, and residential geography. Data on race and ethnicity were included in this study to explore whether there are racial differences by outcomes examined. Additional measures of social marginalization and health care included living at or below the state poverty level, past-year homelessness, lifetime engagement in sex work, lifetime history of incarceration, current insurance status (public, private, or none), past-year mental health treatment (yes, no but needed, or no did not need), current hormone use (yes, no but wanted, or no did not need), substance use problems based on the 4-item Drug Use Disorders Identification Test^26^ (α = 0.96), and harmful alcohol use based on the 4-item Alcohol Use Disorder Identification Test–Concise^27^ (α = 0.63).

### Statistical Analysis

Univariable and bivariable analyses were conducted using proportions and χ^2^ or Fisher exact tests, respectively, and as appropriate, to examine associations of key independent variables (ie, awareness and concerns of antitrans policy environment) and covariates (ie, demographics, social marginalization, and health care experiences) with primary outcomes (ie, current depression and current anxiety). With each primary outcome modeled separately, multivariable regressions were fit and built to model the independent variables (ie, awareness and concerns of the antitrans policy environment) as main exposures while adjusting for all covariates. Interactions between awareness and concerns were included to investigate whether the association of worriedness with mental health symptoms varied according to the knowledge of policy protections. We used a 2-tailed significance testing with an α set to *P* < .05. All analyses were performed using Stata SE statistical software version 16.1 (StataCorp).

## Results

### Sample Characteristics

This cross-sectional study includes a sample of 797 trans Washington adults. A comprehensive overview of demographic, social marginalization, and health care variables is shown in Table 1 by outcome and in Table 2 by exposure. The majority of the participants were young adults (455 individuals aged 18-29 years [57.09%]), were non-Hispanic or Latinx and White (480 individuals [60.30%]), were women (653 women [81.93%]), lived in large or small cities (536 individuals [67.25%]), and had less than a college degree (652 individuals [81.81%]). A total of 10 individuals (1.26%) were American Indian or Alaska Native, 89 (11.18%) were Asian or Asian American, 102 (12.81%) were Black or African American, 69 (8.67%) were Hispanic or Latinx, 7 (0.88%) were Middle Eastern or North African, 11 (1.38%) were Native Hawaiian or Pacific Islander, and 28 (3.52%) reported multiple races or ethnicities. Participants were diverse in terms of sexual orientations.

**Table 1.** Characteristics of Participants by Outcomes Explored

Characteristic	All participants, No. (%) (N = 797 [100.00%])	Depression, current		Anxiety, current	
		Participants, No. (%) (n = 689 [86.45%])	*P* value, χ^2^ test	Participants, No. (%) (n = 686 [86.07%])	*P* value, χ^2^ test
Demographics					
Age, y					
18-24	89 (11.17)	75 (10.89)	<.001	79 (11.52)	.57
25-29	366 (45.95)	327 (47.46)		314 (45.77)	
30-34	285 (35.76)	249 (36.14)		247 (36.01)	
≥35	57 (7.15)	33 (5.52)		46 (6.71)	
Gender identity					
Women	653 (81.93)	578 (83.89)	<.001	575 (83.82)	.003
Men	77 (9.66)	60 (8.71)		59 (8.60)	
Nonbinary, including gender nonconforming	67 (8.41)	51 (7.40)		52(7.58)	
Race and ethnicity					
American Indian or Alaska Native	10 (1.26)	7 (1.02)	.03^a^	8 (1.17)	.005^a^
Asian or Asian American	89 (11.18)	69 (10.01)		69 (10.06)	
Black or African American	102 (12.81)	89 (12.92)		94 (13.70)	
Hispanic or Latinx	69 (8.67)	62 (9.00)		60 (8.75)	
Middle Eastern or North African	7 (0.88)	7 (1.02)		7 (1.02)	
Native Hawaiian or Pacific Islander	11 (1.38)	9 (1.31)		7 (1.02)	
Multiple races or ethnicities	28 (3.52)	21 (3.05)		20 (2.92)	
White, non-Hispanic or Latinx	480 (60.30)	425 (61.68)	.04	421 (61.37)	.12
Sexual orientation					
Another sexual orientation	19 (2.38)	16 (2.33)	<.001^a^	18 (2.61)	.004^a^
Bisexual	67 (8.41)	46 (6.71)		51 (7.40)	
Gay, lesbian, or same-gender attracted	342 (42.91)	313 (45.63)		298 (43.25)	
Queer	66 (8.28)	50 (7.29)		50 (7.26)	
Straight or heterosexual	303 (38.02)	261 (38.05)		272 (39.48)	
Education					
Elementary, high school graduate, or General Educational Development	425 (53.32)	377 (54.72)	.04	366 (53.34)	.04
Some college or technical school	277 (34.76)	237 (34.40)		246 (35.86)	
College or graduate school	95 (11.92)	75 (10.89)		74 (10.79)	
Residential geography					
Large city	308 (38.64)	259 (37.59)	.38	266 (38.78)	.89
Rural area	103 (12.92)	91 (13.21)		89 (12.97)	
Small city or town	228 (28.61)	203 (29.46)		198 (28.61)	
Suburb near a large city	158 (19.82)	136 (19.74)		158 (19.82)	
Social marginalization					
Living below or at state poverty level					
Yes	151 (18.95)	132 (19.16)	.70	127 (18.51)	.44
No	646 (81.05)	557 (80.84)		559 (81.49)	
Homelessness in the past 12 mo					
Yes	24 (3.01)	22 (3.19)	.76^a^	23 (3.35)	.23
No	773 (96.99)	667 (96.81)		663 (96.65)	
Engagement in sex work					
Yes	72 (9.03)	69 (10.01)	.01^a^	68 (9.91)	.03^a^
No	725 (90.97)	620 (89.99)		618 (90.09)	
Incarceration history					
Yes	19 (2.38)	18 (2.61)	>.99^a^	17 (2.48)	>.99^a^
No	778 (97.62)	671 (97.39)		669 (97.52)	
Health care experiences					
Insurance					
Yes, public	167 (20.95)	156 (22.64)	.006^a^	151 (22.01)	.11^a^
Yes, private	626 (78.54)	529 (76.78)		532 (77.55)	
Do not know or no insurance	4 (0.50)	4 (0.58)		3 (0.44)	
Past-year mental health treatment					
Yes, received treatment	90 (11.29)	82 (11.90)	.09^a^	83 (12.10)	<.001^a^
No, but needed treatment	430 (53.95)	363 (52.69)		352 (51.31)	
No, and do not need treatment	255 (31.99)	227 (32.95)		234 (34.11)	
Do not know or not sure	22 (2.76)	17 (2.47)		17 (2.48)	
Currently taking hormones					
Yes	505 (63.36)	444 (64.44)	<.001	443 (64.58)	<.001^a^
No, but wanted	140 (17.57)	104 (15.09)		100 (14.58)	
No, and did not want	152 (19.07)	141 (20.46)		143 (20.85)	
Substance use problems					
Yes	45 (5.65)	45 (6.53)	.002^a^	45 (6.56)	<.001^a^
No	752 (94.35)	644 (93.47)		641 (93.44)	
Harmful alcohol use					
Yes	421 (52.82)	385 (55.88)	<.001^a^	386 (56.27)	<.001^a^
No	376 (47.18)	304 (44.12)		300 (43.73)	
Awareness and concerns of antitrans policy environment					
Knowledge of hate crime protection policy					
Knew incorrectly	460 (57.72)	410 (59.51)	<.001	412 (60.06)	<.001
Did not know or unsure	94 (11.79)	88 (12.77)		88 (12.83)	
Knew correctly	243 (30.49)	191 (27.72)		186 (27.11)	
Concern about taking away rights					
Yes, worried	369 (46.30)	360 (52.25)	.04	348 (50.73)	<.001
No, not worried	428 (53.70)	329 (47.75)		338 (49.27)	

^a^ Two-tailed Fisher exact test was used for cells with fewer than 10 observations.

**Table 2.** Characteristics of Participants by Exposure Variable

Characteristic	All participants, No. (%) (N = 797 [100.00%])	Knowledge of hate crime protection policy (knew correctly) (n = 243 [30.49%])		Concern about taking away rights (yes, worried) (n = 369 [46.30%])	
		Participants, No. (%)	*P* value, χ^2^ test	Participants, No. (%)	*P* value, χ^2^ test
Demographics					
Age, y					
18-24	89 (11.17)	58 (23.87)	<.001	45 (12.20)	.65
25-29	366 (45.95)	128 (52.67)		161 (43.63)	
30-34	285 (35.76)	41 (16.87)		135 (36.59)	
≥35	57 (7.15)	16 (6.58)		28 (7.59)	
Gender identity					
Women	653 (81.93)	145 (59.67)	<.001	300 (81.30)	.85
Men	77 (9.66)	54 (22.22)		38 (10.30)	
Nonbinary, including gender nonconforming	67 (8.41)	44 (18.11)		31 (8.40)	
Race and ethnicity					
American Indian or Alaska Native	10 (1.26)	8 (3.31)	<.001	5 (1.36)	.95
Asian or Asian American	89 (11.18)	21 (8.68)		46 (12.47)	
Black or African American	102 (12.81)	23 (9.50)		45 (12.20)	
Hispanic or Latinx	69 (8.67)	10 (4.13)		32 (8.67)	
Middle Eastern or North African	7 (0.88)	4 (1.65)		4 (1.08)	
Native Hawaiian or Pacific Islander	11 (1.38)	10 (4.13)		6 (1.63)	
Multiple races or ethnicities	28 (3.52)	18 (7.44)		12 (3.25)	
White, non-Hispanic or Latinx	480 (60.30)	148 (61.16)	.01	219 (59.35)	.61
Sexual orientation					
Another sexual orientation	19 (2.38)	10 (4.12)	<.001	10 (2.71)	.002
Bisexual	67 (8.41)	56 (23.05)		37 (10.03)	
Gay, lesbian, or same-gender attracted	342 (42.91)	84 (34.57)		177 (37.97)	
Queer	66 (8.28)	48 (19.75)		32 (8.67)	
Straight or heterosexual	303 (38.02)	45 (18.52)		113 (30.62)	
Education					
Elementary, high school graduate, or General Educational Development	425 (53.32)	95 (39.09)	<.001	172 (46.61)	.002
Some college or technical school	277 (34.76)	89 (36.63)		147 (39.84)	
College or graduate school	95 (11.92)	29 (24.28)		50 (13.55)	
Residential geography					
Large city	308 (38.64)	146 (60.08)	<.001^a^	159 (43.09)	<.001
Rural area	103 (12.92)	0		56 (15.18)	
Small city or town	228 (28.61)	70 (28.81)		74 (20.05)	
Suburb near a large city	158 (19.82)	27 (11.11)		80 (21.68)	
Social marginalization					
Living below or at state poverty level					
Yes	151 (18.95)	79 (32.51)	<.001	61 (16.53)	.11
No	646 (81.05)	164 (67.49)		308 (83.47)	
Homelessness in the past 12 mo					
Yes	24 (3.01)	13 (5.35)	.02^a^	15 (4.07)	.15
No	773 (96.99)	230 (94.65)		354 (95.93)	
Engagement in sex work					
Yes	72 (9.03)	46 (18.93)	<.001	43 (11.65)	.02
No	725 (90.97)	197 (81.07)		326 (88.35)	
Incarceration history					
Yes	19 (2.38)	9 (3.70)	.048^a^	9 (2.44)	.99
No	778 (97.62)	234 (96.30)		360 (97.56)	
Health care experiences					
Insurance					
Yes, public	167 (20.95)	124 (51.03)	<.001^a^	103 (27.91)	<.001
Yes, private	626 (78.54)	115 (47.33)		264 (71.54)	
Do not know or no insurance	4 (0.50)	4 (1.65)		2 (0.54)	
Past-year mental health treatment					
Yes, received treatment	90 (11.29)	53 (21.81)	<.001	59 (15.99)	<.001
No, but needed treatment	430 (53.95)	136 (55.97)		169 (45.80)	
No, and do not need treatment	255 (31.99)	33 (13.58)		131 (35.50)	
Do not know or not sure	22 (2.76)	21 (8.64)		10 (2.71)	
Currently taking hormones					
Yes	505 (63.36)	61 (25.10)	<.001	266 (72.09)	<.001
No, but wanted	140 (17.57)	105 (43.21)		57 (15.45)	
No, and did not want	152 (19.07)	77 (31.69)		46 (12.47)	
Substance use problems					
Yes	45 (5.65)	24 (9.88)	.003	30 (8.13)	.005
No	752 (94.35)	219 (90.12)		339 (91.87)	
Harmful alcohol use					
Yes	421 (52.82)	102 (41.98)	<.001	186 (50.41)	.23
No	376 (47.18)	141 (58.02)		183 (49.59)	

^a^ Two-tailed Fisher exact test was used for cells with fewer than 10 observations.

Approximately 1 in 5 participants (151 individuals [18.95%]) reported living at or below the poverty levels, and less than 10% reported past-year homelessness (24 individuals [3.01%]), lifetime engagement in sex work (72 individuals [9.03%]), or history of incarceration (19 individuals [2.38%]). Most participants reported having public (167 individuals [20.95%]) or private (626 individuals [78.54%]) health insurance, but more than one-half of the participants (430 individuals [53.95%]) reported having unmet need for mental health care.

Most of the participants screened positive for current depression symptoms (689 individuals [86.45%]), as well as current anxiety symptoms (686 individuals [86.07%]). Distribution of current depression and anxiety symptoms by demographic, social marginalization, and health care variables are shown in Table 1. Most of the participants (626 individuals [91.25%]) reported having both current depression and anxiety symptoms (Table 3).

**Table 3.** Sample Distribution by Current Anxiety and Depression Symptoms

Depression, current	Participants, No. (%)		*P* value, χ^2^ test
	Anxiety, current, yes	Anxiety, current, no	
Yes	626 (91.25)	63 (56.76)	<.001
No	60 (8.75)	48 (43.24)	

More than one-half of participants (460 individuals [57.72%]) reported having incorrect knowledge of the current state-level hate crime protection policy, whereas 94 individuals (11.79%) reported not knowing or being unsure. Approximately one-half of the sample (369 individuals [46.30%]) reported being worried about having trans rights taken away. The distribution of state-level hate crime protection policy knowledge and worriedness of trans rights being taken away by demographic, social marginalization, and health care variables is shown in Table 2.

### Main Outcome and Interaction Effect Estimates on Depression

Table 4 displays the results of the multivariable logistic regressions estimating associations of antitrans policy knowledge and concerns with current depression, adjusted for demographics, social marginalization, and health care experiences. Trans adults who were worried about having their rights taken away had significantly higher odds of current depression symptoms (adjusted odds ratio [aOR], 1.66; 95% CI, 1.08-2.54) compared with those who were not worried. Moreover, trans adults with accurate knowledge of state-level protective hate crime policy had significantly lower odds of current depression symptoms (aOR, 0.44; 95% CI, 0.28-0.67), compared with those with inaccurate knowledge.

**Table 4.** Results of Adjusted Multivariable Logistic Regression Separately Modeling Current Depression Symptoms in a Statewide Sample of Transgender Participants^a^

Knowledge and concerns of antitrans policy environment	Depression (current), adjusted OR (95% CI)
Concern about taking away rights	
Yes, worried	1.66 (1.08-2.54)
No, not worried	1 [Reference]
Knowledge of hate crime protection policy	
Knew incorrectly	1 [Reference]
Did not know or unsure	1.92 (0.79-4.64)
Knew correctly	0.44 (0.28-0.67)
Interaction	
No, not worried and knew incorrectly	1.28 (0.70-2.33)
No, not worried and did not know or unsure	1.33 (0.21-8.44)
No, not worried and knew correctly	0.14 (0.05-0.37)
Yes, worried and knew incorrectly	1 [Reference]
Yes, worried and did not know or unsure	0.67 (0.15-2.99)
Yes, worried and knew correctly	0.73 (0.24-2.19)

Abbreviation: OR, odds ratio.

^a^ Model is adjusted for age, gender identity, race and ethnicity (dichotomous), sexual orientation, education, residential geography, poverty level, homelessness, sex work engagement, incarceration history, insurance, past-year mental health treatment, current hormone use, drug dependency, and hazardous drinking. Each outcome was run separately.

When exploring interaction effect estimates, trans adults with accurate knowledge of state-level protective hate crime policy and who were not worried about having their rights taken away reported lower odds of current depression compared with other groups. Specifically, those with accurate policy knowledge who were not worried reported lower odds of current depression (aOR, 0.14; 95% CI, 0.05-0.37) compared with those with incorrect policy knowledge and were worried.

### Main Outcome and Interaction Effect Estimates on Anxiety

Similar associations were observed in the adjusted multivariable logistic regressions estimating current anxiety (Table 5). Trans adults who were worried about having their rights taken away had significantly higher odds of current anxiety symptoms (aOR, 2.67; 95% CI, 1.63-4.36) compared with those who were not worried. Trans adults with accurate knowledge of state-level protective hate crime policy had significantly lower odds of current anxiety symptoms (aOR, 0.11; 95% CI, 0.04-0.25), compared with those with inaccurate knowledge.

**Table 5.** Results of Adjusted Multivariable Logistic Regression Separately Modeling Current Anxiety Symptoms in a Statewide Sample of Transgender Participants^a^

Awareness and concerns of antitrans policy environment	Anxiety (current), adjusted OR (95% CI)
Concern about taking away rights	
Yes, worried	2.67 (1.63-4.36)
No, not worried	1 [Reference]
Knowledge of hate crime protection policy	
Knew incorrectly	1 [Reference]
Did not know or unsure	0.46 (0.13-1.61)
Knew correctly	0.11 (0.04-0.25)
Interaction	
No, not worried and knew incorrectly	0.53 (0.28-1.00)
No, not worried and did not know or unsure	0.48 (0.07-3.07)
No, not worried and knew correctly	0.03 (0.01-0.09)
Yes, worried and knew incorrectly	1 [Reference]
Yes, worried and did not know or unsure	0.18 (0.04-0.85)
Yes, worried and knew correctly	0.22 (0.07-0.67)

Abbreviation: OR, odds ratio.

^a^ Model is adjusted for age, gender identity, race and ethnicity (dichotomous), sexual orientation, education, residential geography, poverty level, homelessness, sex work engagement, incarceration history, insurance, past-year mental health treatment, current hormone use, drug dependency, and hazardous drinking. Each outcome was run separately.

As with depression, trans adults with correct knowledge of state-level protective hate crime policy and who were not worried about having their rights taken away reported lower odds of current anxiety compared with other groups. Specifically, those with correct policy knowledge who were not worried reported lower odds of current anxiety (aOR, 0.03; 95% CI, 0.01-0.09) compared with those with incorrect policy knowledge who were worried. Moreover, among those who were worried about having their trans rights taken away, those with correct knowledge or who were unsure about their knowledge of state-level protective policy reported lower odds of current anxiety (knew correctly, aOR, 0.22; 95% CI, 0.07-0.67; did not know or unsure, aOR, 0.18; 95% CI, 0.04-0.85), compared with those with incorrect knowledge.

## Discussion

This cross-sectional study of trans adults in Washington examined the association between concern regarding the status of trans rights, awareness of legislative protections, and their impact on mental health outcomes in a sample of trans individuals. The findings revealed that trans individuals who were worried about having their rights taken away had significantly higher odds of experiencing depression and anxiety symptoms. Contrarily, those who knew about the state-level protective legislation, specifically protections against hate crimes, had lower odds of depression and anxiety symptoms. Moreover, when examining interaction effect estimates, this study found that trans individuals who correctly knew about the protective policy and were not worried about having their rights taken away reported the lowest odds of depression and anxiety. Taken together, the results highlight the detrimental impact of antitrans legislation and sociopolitical environments on the mental health of trans individuals.

The findings were consistent with prior research^28,29,30,31^ that has documented and elucidated the negative consequences of legislative attacks on the rights of trans people. Although most of those studies relied on policy index mapping to quantify the degree of policy protections, our study supported and further expanded this literature by highlighting the impact of antitrans policy environments as psychologically experienced by trans adults, even in states where trans health is protected. In addition, our research reinforced the results of previous studies^28,30^ that indicated increased levels of depression and anxiety in trans individuals who were concerned about losing their rights, highlighting the psychological impact of living under persistent threat and uncertainty. Moreover, the rates of current depression and anxiety symptoms of this study (reported separately and together) were higher than those previously reported in other studies.^1,2^ This was likely because data from those studies may capture the prevalence of mental health outcomes with a dataset that predates 2021 when antitrans legislation in the US first started escalating.^12,13,14^ Future research should investigate the longitudinal linkages between the prevalence of adverse mental health outcomes and antitrans legislation over time and examine ways to broadly address mental health outcomes.

Although our study highlights the deleterious impact of antitrans policies, it has also provided evidence supporting the positive impact of awareness of protective policies on mental health outcomes in trans people. Specifically, we found that Washingtonian individuals with greater awareness of state-level protective hate crime policy had significantly lower levels of depression and anxiety symptoms than those with less awareness, thereby illuminating the crucial role that awareness of legal protections could play in safeguarding the mental well-being of transgender individuals.^32^ Moreover, in the current study, the interaction effect estimates highlight the importance of psychological processes, whereby those who reported the lowest odds of depression and anxiety symptoms had accurate knowledge about the protective policy and were not worried about their rights being taken away. Even among trans Washingtonians who were worried about their rights being taken away, knowing about the protective policy correctly was also associated with lower odds of current anxiety. Taken together, these findings suggest that accurate knowledge of protective policies, coupled with targeting distress or anxiety around losing trans rights, could buffer the negative impact of antitrans environments on mental health.

The findings provided several implications for research and policy, particularly in designing possible intervention components that could mitigate the negative impact of antitrans legislation. From a social and legal epidemiological research perspective, understanding factors that could contribute to misperceptions or incorrect policy knowledge could help mitigate the impact of inaccurate knowledge on mental health. Research exploring health communication strategies with community members relating to trans rights and state-level protective policies and laws could be a promising mental health intervention.^33,34^ Furthermore, working side-by-side with grassroots trans-led policy organizations in developing content at the local and state levels could help provide awareness and equip trans communities with knowledge and potentially alleviate some of the mental health burdens they face from being exposed to antitrans legislation.^35,36,37^

### Limitations

Some limitations of the study include having a sampling approach that limits the generalizability of the findings to the broader trans populations. Future research should consider sampling in other US states and localities. The findings of this study are also reflective of mostly trans women, with less insights about the mental health of trans men and nonbinary or gender-diverse individuals. We were also unable to conduct intercategorical intersectionality analyses to examine differences by race and ethnicity and other marginalized identities, which is an important area for future research. In addition, the study’s cross-sectional design limits our ability to establish the directionality of the observed associations. Because of the study design, it is possible that there could be inverse directionality between exposure and outcomes. Research on this topic should build on designing longitudinal studies that would provide more robust evidence of directionality and the impact of awareness of legislative protections on mental health outcomes over time. Additional nuanced research questions and design would also be beneficial to extend scope of this study, including delineating how knowledge and concerns about legislation could impact trans individuals reporting anxiety and depression symptoms together or separately. Furthermore, although efforts were made to ensure the survey’s face validity and appropriateness, there may still be measurement limitations. Although the survey items were codeveloped on the basis of the input of community members and experts, a strength of community-engaged research practices,^23^ there are still unmeasured factors that were not captured (eg, the impact of ongoing widespread state-level legislative decisions such as antiabortion laws, which can limit trans people’s bodily autonomy and may impact mental health outcomes^38^).

## Conclusions

This Washington-based study provides valuable insights into legislative protections and mental health outcomes among trans individuals, particularly during the escalated antitrans US sociopolitical climate on trans health care. Important implications for research and policy practices include (1) the need for continued efforts to protect and expand legal rights for trans individuals’ access to health care, (2) prioritizing the importance of education and accurate information communication by equipping trans communities with knowledge of the policy setting that could help counter ambiguity and uncertainty in policy landscape and rights protections, and (3) identifying possible legal and behavioral multilevel intervention components that harness multisectoral partnerships (eg, medical-legal partnerships, community-wide advocacy, and awareness campaigns) that can help counteract, mitigate, or eliminate the negative mental health impact as observed from this study’s findings.

## Notes

Supplement.
Data Sharing Statement
