# Supplementary material for: Antitrans Policy Environment and Depression and Anxiety Symptoms in Transgender and Nonbinary Adults
Source: JAMA Netw Open. 2024 Aug 22;7(8):e2431306. doi: 10.1001/jamanetworkopen.2024.31306 (PMC11342136; doi:10.1001/jamanetworkopen.2024.31306)
Supplement: Supplement. — Data Sharing Statement [file jamanetwopen-e2431306-s001.pdf]

# Data Sharing Statement

Restar. Antitrans Policy Environment and Depression and Anxiety Symptoms in Transgender and Nonbinary Adults. *JAMA Netw Open*. Published August 22, 2024. doi:10.1001/jamanetworkopen.2024.31306

**Data**
**Data available:** No

**Additional Information**
**Explanation for why data not available:** Data is not available for public use due to sensitive information, but can be requested following procedures and approval from the UW IRB Office.
